# Prospective Isolation of Murine and Human Bone Marrow Mesenchymal Stem Cells Based on Surface Markers

**DOI:** 10.1155/2013/507301

**Published:** 2013-05-20

**Authors:** Yo Mabuchi, Diarmaid D. Houlihan, Chihiro Akazawa, Hideyuki Okano, Yumi Matsuzaki

**Affiliations:** ^1^Department of Physiology, Keio University School of Medicine, Shinanomachi 35, Shinjuku-ku, Tokyo 160-8582, Japan; ^2^Department of Biochemistry and Biophysics, Graduate School of Health Care Sciences, Tokyo Medical and Dental University, Yushima 1-5-45, Bunkyo-ku, Tokyo 113-8510, Japan; ^3^Centre for Liver Research, NIHR Biomedical Research Unit, University of Birmingham, Edgbaston, Birmingham B15 2TT, UK; ^4^Institute of Medical Science, Tokyo Medical University, Shinjuku 6-1-1, Shinjuku-ku, Tokyo 160-8402, Japan

## Abstract

Mesenchymal stem cells (MSCs) are currently defined as multipotent stromal cells that undergo sustained *in vitro* growth and can give rise to cells of multiple mesenchymal lineages, such as adipocytes, chondrocytes, and osteoblasts. The regenerative and immunosuppressive properties of MSCs have led to numerous clinical trials exploring their utility for the treatment of a variety of diseases (e.g., acute graft-versus-host disease, Crohn's disease, multiple sclerosis, osteoarthritis, and cardiovascular diseases including heart failure and myocardial infarction). On the other hand, conventionally cultured MSCs reflect heterogeneous populations that often contain contaminating cells due to the significant variability in isolation methods and the lack of specific MSC markers. This review article focuses on recent developments in the MSC research field, with a special emphasis on the identification of novel surface markers for the *in vivo* localization and prospective isolation of murine and human MSCs. Furthermore, we discuss the physiological importance of MSC subtypes *in vivo* with specific reference to data supporting their contribution to HSC niche homeostasis. The isolation of MSCs using selective markers (combination of PDGFR**α** and Sca-1) is crucial to address the many unanswered questions pertaining to these cells and has the potential to enhance their therapeutic potential enormously.

## 1. Introduction

Bone marrow (BM) is comprised of hematopoietic stem cell (HSC) and nonHSC populations. Mesenchymal stem cells (MSCs) reside in the nonHSC fraction. HSCs form the cornerstone of therapy for many hematological diseases. MSCs, on the other hand, are nonhematopoietic cells initially identified in the BM [1–4] that can differentiate along various mesenchymal lineages to generate fat, bone, and cartilage. The hypothesized physiological function of MSCs is to support hematopoiesis and stromal tissue regeneration. Interestingly, these multipotent cells are found in a variety of fetal and adult tissues in addition to the BM, including umbilical cord blood [5, 6], dental pulp [7, 8], term placenta [9, 10], and adipose tissue [11, 12].

**Figure 1:**
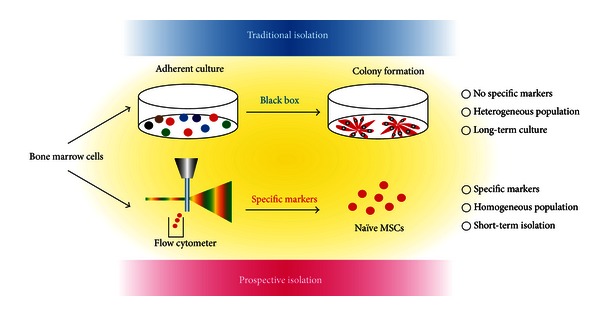
Prospective isolation of MSCs. Traditional MSC isolation by adherent culture on plastic tissue culture substrates (top). Following a period of prolonged culture, the majority of the contaminating cells are washed away or overgrown, enriching for CFU-Fs (colony formation). These MSCs have a spindle-shaped morphology and are capable of differentiating into adipocytes, chondrocytes, and osteoblasts *in vitro*. Prospective isolation of MSCs by using specific markers via flow cytometry (bottom). This method allows the isolation of a pure population of active, multipotent MSCs (naïve MSCs) and avoids cellular contamination.

 MSCs possess therapeutic potential for the repair and regeneration of damaged tissues of mesenchymal origin [13, 14]. Additionally, they have potent immunosuppressive properties and are currently utilized to treat a wide variety of autoimmune conditions [15–19]. Despite the large number of clinical studies now investigating the suitability of MSCs as therapeutic agents, conventional adherence to a plastic tissue culture substrate is still the most commonly employed method for their isolation. However, isolating MSCs in this way has several limitations. For example, such MSC populations frequently contain contaminating cells. Furthermore, the differentiation potential and proliferative ability of traditionally isolated MSCs (also termed colony forming unit-fibroblasts (CFU-Fs)) gradually diminish as the cells mature [20]. MSCs may also acquire chromosomal abnormities that predispose them to malignant transformation [21]. Finally, prolonged culture on plastic dishes changes the surface marker expression of MSCs, making identification of selective makers difficult [22, 23]. For these reasons, little information exists concerning the *in vivo* identity and biological function of MSCs within the BM niche. Nonetheless, exciting progress has recently been made in terms of elucidating reliable murine and human MSC surface markers offering exciting experimental and therapeutic opportunity (Figure 1).

 This review summarizes the historical identification of MSCs and important milestones in the evolution of MSC research. We focus on the identification of MSCs in mouse and human and describe the utilization of specific murine and human MSC surface markers to facilitate the *in vivo* localization and prospective isolation of these cells. Finally, we summarize the evidence supporting a physiological role for MSCs within the BM/hematopoietic niche.

## 2. Historical Perspective

Dr. Friedenstein initially identified BM-derived, plastic-adherent cells that generated CFU-Fs when plated as single cells *in vitro* [24, 25]. Dr. Friedenstein subsequently demonstrated that these cells were capable of osteogenic differentiation *in vitro*. The physiological function of MSCs was next elegantly demonstrated by Reddi and colleagues, who subcutaneously implanted biological matrices comprising the shafts of long bones into allogenic rodents [26]. Bone and cartilage formed on the implants after a period of time, and the resulting bony ossicle supported hematopoiesis *in vivo*. These data were the first to support the presence of stromal progenitors and to illustrate their biological significance. Largely based on these studies, the term “MSC” was coined in 1991 to describe stromal progenitor cells [27]. Although MSCs have since become the subject of intense research, very little was uncovered until recently in regard to their anatomical localization, physiological function, and stromal hierarchy [28].

## 3. Definition of MSCs

Traditionally, MSCs appear as spindle-shaped cells that form colonies (i.e., CFU-Fs) following the culture of whole BM on plastic substrates. The multilineage potential of these colonies is then examined after a period of culture in defined media that induces cell differentiation. Additionally, phenotypic analysis of MSCs is determined by their culture conditions. Therefore, MSC properties have historically been described for plastic-adherent cells after prolonged* in vitro *culture. Although conventionally cultured MSCs are not characterized by unique markers and probably denote a heterogeneous population, there is a consensus among the scientific community that they do not express hematopoietic markers. Hence, MSCs stand apart from HSCs. Furthermore, the expression levels of stromal antigens in MSCs can vary based on the culture conditions. The Tissue Stem Cell Committee of the International Society for Cellular Therapy thus proposed a set of minimum criteria that define human MSCs [29] as follows. The cell must be plastic-adherent when cultured under standard conditions and express the surface markers cluster of differentiation (CD) 73, CD90, and CD105, and not express CD45, CD34, CD14, CD11b, CD79, or CD19. Additionally, human MSCs must be capable of *in vitro* differentiation into osteoblasts, adipocytes, and chondrocytes. 

 While this statement somewhat clarifies the cellular characteristics of human MSCs, the situation remains unclear for murine MSCs. Until recently, specific surface markers for murine MSCs were lacking and murine MSCs were also defined by plastic adherence, spindle-shaped morphology, and trilineage differentiation. These definitions for MSC isolated from both species have however generated controversy. The classic definition of a stem cell requires that it possess unlimited self-renewal ability and plasticity. Experimentally, serial transplantation experiments demonstrating that infused stem cells give rise to terminally differentiated daughter cells, while maintaining their naïve phenotype, provide evidence of stemness. Such experiments were not historically performed with MSCs, leading researchers to consider that the term “MSC” had been inappropriately applied [30]. 

## 4. Identification of Specific Murine MSC Markers

The identification of specific murine MSC markers began with the observation that hematopoietic and mesenchymal lineage cells are derived from individual lineage-specific stem cells [31]. Based on the hypothesis that MSCs most likely reside in the endosteum, a detailed screening of candidate surface markers was initially performed in the BM and the collagenase-digested bone of mice. The surface markers, platelet-derived growth factor receptor-*α* (PDGFR-*α*), and stem cell antigen-1 (Sca-1) were significantly enriched in the digested fraction of the bone, and PDGFR-*α* ^+^Sca-1^+^ (P*α*S) dual positive cells were isolated and characterized [32, 33]. Notably, the resultant P*α*S cells fulfill the basic requirements for the definition of MSCs in mice. These cells are capable of unlimited self-renewal and can differentiate into osteoblasts, chondrocytes, and adipocytes under appropriate conditions *in vitro *[33]. P*α*S cells proliferate almost without senescence when cultured on plastic, yielding more than 1 × 10^7^ cells from an original 5,000 cells seeded onto the substrate, with a doubling time of 50.6 hours. Moreover, the CFU-F frequency of P*α*S cells is approximately 120,000-fold higher than that of unfractionated BM mononuclear cells. 

P*α*S cells reside in the perivascular space adjacent to vascular smooth muscle in mice. They express angiopoietin-1 (Ang-1) and chemokine (C-X-C motif) ligand 12 (CXCL12), suggesting that these MSCs play a physiological role in the maintenance of the hematopoietic niche. Transplantation experiments in which freshly isolated P*α*S cells were intravenously injected into lethally irradiated recipient mice demonstrated the stemness of P*α*S cells. Specifically, the infused cells homed to their niche in the BM and continued to express the hematopoietic niche factors Ang-1 and CXCL12, while also differentiating into osteoblasts and adipocytes *in vivo*. Sixteen weeks following cell transplantation, the mice were sacrificed, and the P*α*S cells were isolated. Notably, the isolated cells were still capable of both CFU-F formation and trilineage differentiation *in vitro*. 

The identification of PDGFR-*α* as a selective MSC marker coupled with Cre/loxP-mediated lineage analysis [34] suggests that a subpopulation of adult BM MSCs might have a developmental origin in the murine neural crest [35, 36]. This is in agreement with a series of previously reported developmental studies in quail, chick, and rat [37, 38]. Murine and human MSCs are also an excellent cell source for the efficient generation of high-quality induced pluripotent stem cells [39, 40], which can in turn generate neural crest-like cells. More recently, a transgenic mouse reporter line expressing GFP under the control of enhancer/promoter of *nestin* gene, encoding an intermediate protein highly expressed in the neural stem/progenitor cells [41], was successfully used to identify and prospectively isolate murine MSCs in BM [42]. The *nestin-*GFP^+^ cells in this transgenic mouse also expressed the intermediate filament protein Nestin and represented a small subset of nonhematopoietic stromal cells in the BM. These cells are anatomically located in the perivascular space, in close proximity to catecholaminergic nerve fibers and HSCs. In keeping with P*α*S MSCs, the *nestin-*GFP^+^ cells express hematopoietic niche factors and are capable of trilineage differentiation both *in vitro* and *in vivo*. Nestin^+^ MSCs also play an important functional role in maintaining the HSC niche. For example, the number of HSCs was dramatically reduced *in vivo* following the depletion of *nestin-*GFP^+^ MSCs in mice, and the homing of transplanted HSCs back to their BM niche was significantly impaired in these animals following irradiation. 

The studies discussed above are the first to identify specific markers that can be used for the *in vivo* localization and prospective isolation of MSCs. P*α*S and Nestin^+^ MSCs have been analyzed in traditional stem cell assays (e.g., serial transplantation assays and clonogenic assays), which confirmed their properties of self-renewal and potency. Our own research group has also gained valuable insights into the importance of these cells in maintaining the HSC niche as well as the possibility of MSC subpopulations within the BM. For example, it is not entirely clear if Nestin^+^ cells are the same as P*α*S cells. We know that Nestin^+^ cells largely overlap with the PDGFR*α* ^+^CD51^+^ population; however, this population also contains Sca-1^+^ and Sca-1^−^ cells (personal communication). These data suggest that the Nestin^+^ population comprises both P*α*S and PDGFR*α* ^+^ cells. Notably, investigations using *nestin-Cherry* [43] and *nestin-GFP* [44] double transgenic mice detected *nestin-Cherry* expression around the larger blood vessels in the BM but not around the sinusoids, while *nestin-GFP* expression was detected around both structures [45]. Thus, different *nestin* promoter/enhancer-driven transgenes are apparently expressed by different subpopulations of perivascular stromal cells. Regardless, the identification and prospective isolation of P*α*S and Nestin^+^ cells will provide indispensable information for ongoing research into the biological function, stromal hierarchy, and therapeutic potential of MSCs.

## 5. Identification of Specific Human MSC Markers

Numerous putative human MSC surface markers (i.e., CD49a [23], CD73 [1], CD105 [46], CD106 [47], CD271 [22], MSC antigen-1 [48], Stro-1 [49], and stage-specific embryonic antigen-4 [50]) have been identified thus far. These markers are used singly or in combination to enrich for CFU-Fs in human BM and avoid cellular contamination. Unfortunately, many of these markers are widely expressed in stromal cells and lack specificity, contributing to the significant heterogeneity among CFU-Fs derived from single isolations. The lack of specific MSC markers has thwarted attempts to uncover the true identity and function of these stem cells *in vivo*. Additionally, the traditional isolation of human MSCs by adherence to plastic substrates attenuates the differentiation potential and proliferative ability of CFU-Fs as the cells senesce, greatly reducing their therapeutic potential [51].

Various techniques, such as culture under hypoxic conditions, culture under nonadherent conditions, and supplementation of the culture media with growth factors, have been used in an attempt to avoid cellular senescence and enhance the therapeutic properties of MSCs. For example, human MSCs cultured as three-dimensional spheroids in a model of peritonitis acquired enhanced anti-inflammatory properties compared with those cultured under more conventional conditions [52]. The spheroid-associated cells were also smaller, allowing them to escape readily from the lung circulation and migrate to a variety of organs after intravenous administration to mice. Other investigators showed that long-term culture of MSCs under hypoxic conditions helps to keep the cells in an undifferentiated and multipotent state [53, 54]. 

As far as clinical applications are concerned, the number of clinical trials using *ex vivo* expanded stromal cell populations for therapeutic purposes is rapidly increasing (see http://www.clinicaltrials.gov/) [55, 56]. For example, MSCs have shown promise for the treatment of acute graft-versus-host disease, Crohn's disease, multiple sclerosis, osteoarthritis, and cardiovascular diseases. However, there is little consistency in the methods used to isolate MSCs for infusion, or in the media used to expand these cells in culture. Commercially available MSC medium frequently contains growth factors (required for cell expansion) that most likely influence the fate and therapeutic potential of the MSCs. These limitations further underscore the need to identify specific surface markers that can be used to probe the physiological functions and biological properties of human MSCs expeditiously. The prospective isolation and culture of such cells (with or without further manipulation) will certainly allow for safer and more effective clinical treatments in the future. 

CD146 is one such marker that has helped discern the *in vivo* localization and function of human MSCs [57]. CD146 is found on the surface of adventitial reticular cells that reside in the endothelial space in human BM. These cells also express typical stromal markers (CD105, CD49a, CD73, CD90, and CD140b) and are capable of robust trilineage differentiation. Their physiological function was shown in immunodeficient mice following subcutaneous transplantation of human CD146^+^ clonogenic cells seeded onto a scaffold (hydroxyapatite/tricalcium phosphate particles embedded in a fibrin gel). The transplanted human CD146^+^ MSCs supported formation of bony ossicles and sinusoidal vasculature and finally established a functioning hematopoietic microenvironment. Immunohistochemical analysis demonstrated that a small proportion of the infused cells targeted the murine HSC niche, where they expressed Ang-1 and other supporting factors. The transplanted human CD146^+^ MSCs were reisolated, cultured, and subsequently shown to form CFU-Fs capable of trilineage differentiation, demonstrating the self-renewal potency of these cells.

MSCs were initially thought to reside only within the BM, forming the stromal counterpart to HSCs. However, the utility of CD146 as a prospective marker for human MSCs is not limited to adult human BM, casting doubt on this assumption. Crisan et al. [58] used immunohistochemistry to examine various tissue types (e.g., adult and fetal human skeletal muscles, pancreas, adipose tissue, and placenta) and identified CD146, neuron-glial antigen 2 proteoglycan, and PDGFR*α* as specific pericyte markers [58]. With the aid of these markers, a pure population of pericytes was prospectively isolated from each tissue type via flow cytometry. The isolated pericytes expressed typical stromal markers (CD73, CD90, and CD105) and could be induced to differentiate into muscle, bone, fat, and cartilage by using standard MSC culture conditions and the appropriate differentiation factors. These data clearly identify CD146 as a specific surface marker of mesenchymal progenitor cells in a wide range of organs.

## 6. Role of MSCs *In Vivo*

The HSC niche provides a specialized microenvironment that promotes stem cell maintenance and function [59–63]. Several cells types, including osteoblasts, endothelial cells, and adventitial reticular cells, have been suggested to contribute to niche function [59, 64]. For many years, MSCs were surmised to be among these cells, although until recently, their participation has remained merely speculative. Our previous observations that P*α*S cells reside in the HSC niche (the perivascular space adjacent to HSCs) and express niche factors (Ang-1 and CXCL12) support the hypothetical involvement of MSCs in the regulation of the HSC microenvironment [33]. Indeed, Nestin^+^ MSCs apparently play a critical role not only in the maintenance of HSCs within the niche, but also in the homing of transplanted HSCs back to the BM. 

Although a significant proportion of the perivascular PDGFR*α* ^+^ cells described above express Nestin, the exact impact of each subpopulation of perivascular cells on the HSC niche remains to be elucidated. Recent data suggest that nonmyelinating Schwann cells participate in the maintenance of the HSC niche via activation of latent transforming growth factor-*β* [65]. It is noteworthy that these cells express Nestin, thus evoking some controversy in the research field as to their possible stemness. Recently, Ding et al. [45] confirmed the importance of perivascular cells in maintaining the HSC niche through the production of stem cell factor (Scf). HSC frequency and function were not affected when Scf was conditionally deleted from hematopoietic cells, osteoblasts, or Nestin^+^ cells. However, HSCs were eliminated from the BM when Scf was deleted from endothelial cells or Leptin receptor-expressing perivascular stromal cells (which were also positive for PDGFR*α*, PDGFR*β*, CXCL12, and alkaline phosphatase expression). Clearly, much remains unknown about the complex microenvironment of the HSC niche and its regulatory factors. Nevertheless, the data suggest that one or more MSC subtypes critically contribute to HSC niche homeostasis.

## 7. Conclusions

The hypothesis that a rare population of multipotent stromal progenitor cells or MSCs, capable of generating all stromal cell subtypes, existed in the BM was greeted with almost universal approval in the scientific world. However, until recently little evidence supported the proposed physiological functions of MSCs, including maintenance of the HSC niche, replenishment of mesenchymal tissue, wound healing, and tissue repair. An absence of specific MSC surface markers proved to be a significant stumbling block to unraveling the biology and function of MSCs. Nonetheless, the field has lately taken a significant leap forward with the identification of such markers in the mouse and human, allowing the prospective isolation of MSCs for the first time. As a result, we can now convincingly assay and confirm the stem cell properties of MSCs and elucidate their biological functions (their role in the maintenance of the HSC niche). We suggest that the prospective isolation (e.g., combination of PDGFR*α* and Sca-1) of MSCs will also allow scientists to address the many unanswered questions related to these cells, and most importantly, to advance MSCs as a therapeutic agent.

## References

[1] Pittenger MF, Mackay AM, Beck SC (1999). Multilineage potential of adult human mesenchymal stem cells. *Science*.

[2] Piersma AH, Brockbank KGM, Ploemacher RE (1985). Characterization of fibroblastic stromal cells from murine bone marrow. *Experimental Hematology*.

[3] Kuznetsov SA, Friedenstein AJ, Robey PG (1997). Factors required for bone marrow stromal fibroblast colony formation *in vitro*. *British Journal of Haematology*.

[4] Conget PA, Minguell JJ (1999). Phenotypical and functional properties of human bone marrow mesenchymal progenitor cells. *Journal of Cellular Physiology*.

[5] Erices A, Conget P, Minguell JJ (2000). Mesenchymal progenitor cells in human umbilical cord blood. *British Journal of Haematology*.

[6] Lee OK, Kuo TK, Chen WM, Lee KD, Hsieh SL, Chen TH (2004). Isolation of multipotent mesenchymal stem cells from umbilical cord blood. *Blood*.

[7] Gronthos S, Mankani M, Brahim J, Robey PG, Shi S (2000). Postnatal human dental pulp stem cells (DPSCs) *in vitro* and *in vivo*. *Proceedings of the National Academy of Sciences of the United States of America*.

[8] Shi S, Gronthos S (2003). Perivascular niche of postnatal mesenchymal stem cells in human bone marrow and dental pulp. *Journal of Bone and Mineral Research*.

[9] Yen BL, Huang HI, Chien CC (2005). Isolation of multipotent cells from human term placenta. *Stem Cells*.

[10] Battula VL, Treml S, Abele H, Bühring HJ (2008). Prospective isolation and characterization of mesenchymal stem cells from human placenta using a frizzled-9-specific monoclonal antibody. *Differentiation*.

[11] Orbay H, Tobita M, Mizuno H (2012). Mesenchymal stem cells isolated from adipose and other tissues: basic biological properties and clinical applications. *Stem Cells International*.

[12] Liu TM, Martina M, Hutmacher DW, Hui JHPO, Eng HL, Lim B (2007). Identification of common pathways mediating differentiation of bone marrow- and adipose tissue-derived human mesenchymal Stem Cells into three mesenchymal lineages. *Stem Cells*.

[13] Stappenbeck TS, Miyoshi H (2009). The role of stromal stem cells in tissue regeneration and wound repair. *Science*.

[14] De Bari C, Dell’Accio F, Vandenabeele F, Vermeesch JR, Raymackers JM, Luyten FP (2003). Skeletal muscle repair by adult human mesenchymal stem cells from synovial membrane. *Journal of Cell Biology*.

[15] Liu Y, Wang L, Kikuiri T (2011). Mesenchymal stem cell-based tissue regeneration is governed by recipient T lymphocytes via IFN-gamma and TNF-alpha. *Nature Medicine*.

[16] Zhao S, Wehner R, Bornhäuser M, Wassmuth R, Bachmann M, Schmitz M (2010). Immunomodulatory properties of mesenchymal stromal cells and their therapeutic consequences for immune-mediated disorders. *Stem Cells and Development*.

[17] Spaggiari GM, Capobianco A, Becchetti S, Mingari MC, Moretta L (2006). Mesenchymal stem cell-natural killer cell interactions: evidence that activated NK cells are capable of killing MSCs, whereas MSCs can inhibit IL-2-induced NK-cell proliferation. *Blood*.

[18] Djouad F, Plence P, Bony C (2003). Immunosuppressive effect of mesenchymal stem cells favors tumor growth in allogeneic animals. *Blood*.

[19] Yañez R, Lamana ML, García-Castro J, Colmenero I, Ramírez M, Bueren JA (2006). Adipose tissue-derived mesenchymal stem cells have *in vivo* immunosuppressive properties applicable for the control of the graft-versus-host disease. *Stem Cells*.

[20] Digirolamo CM, Stokes D, Colter D, Phinney DG, Class R, Prockop DJ (1999). Propagation and senescence of human marrow stromal cells in culture: a simple colony-forming assay identifies samples with the greatest potential to propagate and differentiate. *British Journal of Haematology*.

[21] Ben-David U, Mayshar Y, Benvenisty N (2011). Large-scale analysis reveals acquisition of lineage-specific chromosomal aberrations in human adult stem cells. *Cell Stem Cell*.

[22] Quirici N, Soligo D, Bossolasco P, Servida F, Lumini C, Deliliers GL (2002). Isolation of bone marrow mesenchymal stem cells by anti-nerve growth factor receptor antibodies. *Experimental Hematology*.

[23] Boiret N, Rapatel C, Veyrat-Masson R (2005). Characterization of nonexpanded mesenchymal progenitor cells from normal adult human bone marrow. *Experimental Hematology*.

[24] Friedenstein AJ, Deriglasova UF, Kulagina NN (1974). Precursors for fibroblasts in different populations of hematopoietic cells as detected by the *in vitro* colony assay method. *Experimental Hematology*.

[25] Friedenstein AJ, Petrakova KV, Kurolesova AI, Frolova GP (1968). Heterotopic of bone marrow.Analysis of precursor cells for osteogenic and hematopoietic tissues. *Transplantation*.

[26] Reddi AH, Huggins CB (1975). Formation of bone marrow in fibroblast transformation ossicles. *Proceedings of the National Academy of Sciences of the United States of America*.

[27] Caplan AI (1991). Mesenchymal stem cells. *Journal of Orthopaedic Research*.

[28] Bianco P, Cao X, Frenette PS (2013). The meaning, the sense and the significance: translating the science of mesenchymal Stem Cells into medicine. *Nature Medicine*.

[29] Dominici M, Le Blanc K, Mueller I (2006). Minimal criteria for defining multipotent mesenchymal stromal cells. The International Society for Cellular Therapy position statement. *Cytotherapy*.

[30] Bianco P, Robey PG, Simmons PJ (2008). Mesenchymal stem cells: revisiting history, concepts, and assays. *Cell Stem Cell*.

[31] Koide Y, Morikawa S, Mabuchi Y (2007). Two distinct stem cell lineages in murine bone marrow. *Stem Cells*.

[32] Houlihan DD, Mabuchi Y, Morikawa S (2012). Isolation of mouse mesenchymal stem cells on the basis of expression of Sca-1 and PDGFR-alpha. *Nature Protocols*.

[33] Morikawa S, Mabuchi Y, Kubota Y (2009). Prospective identification, isolation, and systemic transplantation of multipotent mesenchymal stem cells in murine bone marrow. *Journal of Experimental Medicine*.

[34] Nagoshi N, Shibata S, Kubota Y (2008). Ontogeny and multipotency of neural crest-derived stem cells in mouse bone marrow, dorsal root ganglia, and whisker pad. *Cell Stem Cell*.

[35] Morikawa S, Mabuchi Y, Niibe K (2009). Development of mesenchymal stem cells partially originate from the neural crest. *Biochemical and Biophysical Research Communications*.

[36] Takashima Y, Era T, Nakao K (2007). Neuroepithelial cells supply an initial transient wave of MSC differentiation. *Cell*.

[37] Le Lievre CS, Le Douarin NM (1975). Mesenchymal derivatives of the neural crest: analysis of chimaeric quail and chick embryos. *Journal of Embryology and Experimental Morphology*.

[38] Morrison SJ, White PM, Zock C, Anderson DJ (1999). Prospective identification, isolation by flow cytometry, and *in vivo* self-renewal of multipotent mammalian neural crest stem cells. *Cell*.

[39] Niibe K, Kawamura Y, Araki D (2011). Purified mesenchymal stem cells are an efficient source for iPS cell induction. *PLoS ONE*.

[40] Wang Y, Liu J, Tan X Induced pluripotent stem cells from human hair follicle mesenchymal stem cells.

[41] Lendahl U, Zimmerman LB, McKay RDG (1990). CNS stem cells express a new class of intermediate filament protein. *Cell*.

[42] Méndez-Ferrer S, Michurina TV, Ferraro F (2010). Mesenchymal and haematopoietic stem cells form a unique bone marrow niche. *Nature*.

[43] Tronche F, Kellendonk C, Kretz O (1999). Disruption of the glucocorticoid receptor gene in the nervous system results in reduced anxiety. *Nature Genetics*.

[44] Mignone JL, Kukekov V, Chiang AS, Steindler D, Enikolopov G (2004). Neural stem and progenitor cells in Nestin-GFP transgenic mice. *Journal of Comparative Neurology*.

[45] Ding L, Saunders TL, Enikolopov G, Morrison SJ (2012). Endothelial and perivascular cells maintain haematopoietic stem cells. *Nature*.

[46] Aslan H, Zilberman Y, Kandel L (2006). Osteogenic differentiation of noncultured immunoisolated bone marrow-derived CD105^+^ cells. *Stem Cells*.

[47] Gronthos S, Zannettino ACW, Hay SJ (2003). Molecular and cellular characterisation of highly purified stromal stem cells derived from human bone marrow. *Journal of Cell Science*.

[48] Battula VL, Treml S, Bareiss PM (2009). Isolation of functionally distinct mesenchymal stem cell subsets using antibodies against CD56, CD271, and mesenchymal stem cell antigen-1. *Haematologica*.

[49] Simmons PJ, Torok-Storb B (1991). Identification of stromal cell precursors in human bone marrow by a novel monoclonal antibody, STRO-1. *Blood*.

[50] Gang EJ, Bosnakovski D, Figueiredo CA, Visser JW, Perlingeiro RCR (2007). SSEA-4 identifies mesenchymal stem cells from bone marrow. *Blood*.

[51] Kim J, Kang JW, Park JH (2009). Biological characterization of long-term cultured human mesenchymal stem cells. *Archives of Pharmacal Research*.

[52] Bartosh TJ, Ylöstalo JH, Mohammadipoor A (2010). Aggregation of human mesenchymal stromal cells (MSCs) into 3D spheroids enhances their antiinflammatory properties. *Proceedings of the National Academy of Sciences of the United States of America*.

[53] Basciano L, Nemos C, Foliguet B (2011). Long term culture of mesenchymal stem cells in hypoxia promotes a genetic program maintaining their undifferentiated and multipotent status. *BMC Cell Biology*.

[54] Jin Y, Kato T, Furu M (2010). Mesenchymal stem cells cultured under hypoxia escape from senescence via down-regulation of p16 and extracellular signal regulated kinase. *Biochemical and Biophysical Research Communications*.

[55] Wang Y, Han ZB, Song YP, Han ZC (2012). Safety of mesenchymal stem cells for clinical application. *Stem Cells International*.

[56] Jackson WM, Nesti LJ, Tuan RS (2012). Concise review: clinical translation of wound healing therapies based on mesenchymal stem cells. *Stem Cells Translational Medicine*.

[57] Sacchetti B, Funari A, Michienzi S (2007). Self-renewing osteoprogenitors in bone marrow sinusoids can organize a hematopoietic microenvironment. *Cell*.

[58] Crisan M, Yap S, Casteilla L (2008). A perivascular origin for mesenchymal stem cells in multiple human organs. *Cell Stem Cell*.

[59] Kiel MJ, Morrison SJ (2006). Maintaining hematopoietic stem cells in the vascular niche. *Immunity*.

[60] Arai F, Hirao A, Ohmura M (2004). Tie2/angiopoietin-1 signaling regulates hematopoietic stem cell quiescence in the bone marrow niche. *Cell*.

[61] Xie Y, Yin T, Wiegraebe W (2009). Detection of functional haematopoietic stem cell niche using real-time imaging. *Nature*.

[62] Köhler A, Schmithorst V, Filippi MD (2009). Altered cellular dynamics and endosteal location of aged early hematopoietic progenitor cells revealed by time-lapse intravital imaging in long bones. *Blood*.

[63] Lo Celso C, Fleming HE, Wu JW (2009). Live-animal tracking of individual haematopoietic stem/progenitor cells in their niche. *Nature*.

[64] Wilson A, Trumpp A (2006). Bone-marrow haematopoietic-stem-cell niches. *Nature Reviews Immunology*.

[65] Yamazaki S, Ema H, Karlsson G (2011). Nonmyelinating Schwann cells maintain hematopoietic stem cell hibernation in the bone marrow niche. *Cell*.

